# Program to Promote Personal and Social Responsibility in the Secondary Classroom

**DOI:** 10.3389/fpsyg.2017.00809

**Published:** 2017-05-22

**Authors:** Miguel A. Carbonero, Luis J. Martín-Antón, Lourdes Otero, Eugenio Monsalvo

**Affiliations:** ^1^Department of Psychology, Excellence Research Group GR179 Educational Psychology, University of ValladolidValladolid, Spain; ^2^Department of Philosophy, University of ValladolidValladolid, Spain; ^3^Instituto de Enseñanza Secundaria Delicias, Junta de Castilla y LeónValladolid, Spain

**Keywords:** social responsibility attitudes, personal attitudes, reasoned-action, mentoring, secondary education

## Abstract

The performance of school children has been studied by considering partial relationships between several personal variables such as the link between cognition and motivation. However, contextual variables, such as a child’s willingness to accept social responsibility, also influence students’ social and academic performance. Thus, students with greater responsibility have a better attitude toward their studies, resulting in higher academic achievement. This 2-year study aims to reveal to what extent an intervention program affects student performance and is based on the Theory of Positive Action among young people proposed by Don Hellison and the Theory of Reasoned Action by Fishbein and Ajzen. The program focuses on positive influences on social and personal responsibility, taking into consideration parental styles, gender, and academic performance. The program was a part of the educational curricula in participating schools and it targeted four main areas: (a) teaching units using academic texts about social responsibility, (b) student training in mediation processes, (c) teacher training, and (d) family training and involvement. A total of 271 students took part from first and second year of Secondary Education (12–14 years old). The experimental group was made up of 132 students while the remaining 139 formed the control group. All participants completed the Assessment Scale of Social Responsibility Attitudes in Secondary Education and the Parent–Adolescent Communication Scale. Results show that students in the experimental group performed significantly better than those in the control group. Additionally, the issue of social responsibility seems to be related to commitment, self-discipline and perseverance. Regarding gender, males appear to score higher in the factor for well-mannered, friendly and tidy. Finally, a positive relationship has been identified between social responsibility attitudes and parenting with an open communicational style. This paper discusses the results so that schools can include programs aimed at improving social and personal responsibility.

## Introduction

Responsibility is the ability that people possess to respond effectively and adequately toward their behaviors, in a way that the person adjusts to those norms that set all social behaviors (Barberá, 2001). Schools and families are expected to be responsible for educating students in this value (Ochs and Izquierdo, 2009; Paradise and De Haan, 2009; Wood et al., 2009; Maliki et al., 2010; Thomas, 2011; Wray-Lake and Syvertsen, 2011; Salusky et al., 2014). In the school and education system, any pedagogical methods will depend on what philosophical and psychological conceptions are used, whereas the promotion of personal and social responsibility in the family will depend on the style of parenting.

From a philosophical point of view, responsibility should be approached as a primary value as Ingarden (1959) suggested. In this view, responsibility would be thought of as a value with a universal and necessary cognitive base, or as a value dependent on cultural conditions and historical facts such as Aristotle and the Hermeneutic Philosophy propose. In line with this author’s approach, responsibility does not relate to knowledge but is rather a characteristic that embodies certain actions. These actions or behaviors could be described as deliberate acts that are carried out using adequate and correct tools in order to achieve aims that, in certain situations, are considered appropriate and correct. Taking this approach into consideration, a person acquires responsibility by the internalization of external norms, along with an adequate development of their cognitive and evaluative ability (Barberá, 2001). The latter capability relates to the theoretical perspective of practical reason rather than theoretical reason, as stated by several authors (Gadamer, 2006; Gadamer and DaVia, 2015). This research follows such an approach. To be precise, it considers the concept of responsibility from Ricoeur’s theoretical line along with the Theory of Narrative Hermeneutics (Ricoeur, 2012; Savage, 2015). According to Ricoeur, people’s narratives prefigure their idea about the world. Considered from this perspective, when people read, they do not only configure the sense of the novels or texts, but they project and imagine their future behavior when similar situations appear in their lives. Therefore, in certain situations, novels could be thought of as ‘labs to take ethical decisions.’ This drives us to form our own idea of the world and the values that help us to define it, which means our vision of the world and the values that help us to define it. Furthermore, this philosophical theory is closely related to Seligman’s proposal about Positive Psychology (Seligman and Csikszentmihalyi, 2000; Seligman et al., 2005; Seligman, 2012) and its ‘character strengths’ (Seligman and Petterson, 2004), where happiness is considered to be a concept that helps to give sense to life.

From a psychological standpoint, the Theory of Moral Development (Kohlberg et al., 1984) and the Theory of Moral Socialization (Hoffman, 2000, 2001) have so far been the main approaches in the area. These theoretical approaches follow different principles. While the Theory of Moral Development is based on Cognitive Psychology proposed by Piaget, the Theory of Moral Socialization takes its principles from the Psychology of Learning. In the first approach, moral development implies a continuous construction of morality (Colby and Kohlberg, 1987), meaning that the person who has achieved an internal or autonomous orientation has a mature comprehension of moral norms and values. Seen from Learning Psychology, moral development would be the transfer of moral norms and values from the society to the child.

From an anthropological point of view, studies carried out by Ochs and Izquierdo (2009), which look at how children give explanations to their families about their daily activities in four different cultures, identify three observed situations when acquiring personal autonomy through the activities of responsibility offered by their parents. The first dimension is social awareness, the second dimension in social sensitivity, and the third dimension, self-sufficiency. Similar studies have been conducted in different countries and cultures such as Nigeria (Maliki et al., 2010), Norway (Bjerke, 2011), Vietnam (Zaharim et al., 2013), and Peru (Paradise and De Haan, 2009; Ames, 2013).

Another psychological approach stems from Symbolic Interaction Theory (SIT, Beranek and Butler, 2006). This theory explains the formation of responsibility taking into consideration the idea that people have a concept of themselves in relation to the interaction with the image that they receive from others. These studies complement the research carried out by Cook and Douglas (1998) who stated that symbolism in the acquisition of responsibility by children consisted in satisfying their role as children, while at the same time keeping their sense of self. Parents act as helpers establishing expectations of each member of the family in order to make the family work. These expectations are transmitted to the school, which makes the teachers behave like parents. These studies confirmed the conclusions obtained by Such and Walker (2004), who suggested that responsibility is a key concept in policy and public debate on the lives of children and their families. On the one hand, parents help children to take responsibility for their welfare and, on the other, children and young people are frequently blamed and punished for “irresponsible” or antisocial behavior. Such and Walker (2004) conclude that from the point of view of children we must make a distinction between ‘doing things in a responsible way’ and ‘doing responsible things.’ Doing things in a responsible way (meaning with common sense, maturity, and trust) would be a way of accessing more responsible things (choosing when and how to do homework, staying alone at home, etc.). For many children, this means “power and autonomy” (Such and Walker, 2004, p. 240). Lister (2008) confirms these findings by adding the need adults have for “acceptance of the child as a responsible human being,” within the social community where he or she lives. Nevertheless, this acceptance would partly depend on the ability of the child to demonstrate their capability to do things in a responsible manner. The combination of being responsible and doing things in a responsible way is the idea that has led other authors (Ochs and Izquierdo, 2009; Thomas, 2011) to propose a theoretical approach toward the acquisition of autonomy and responsibility that could be framed within Self-Determination Theory (STD, Deci and Ryan, 2000, 2012). This theory states that there are three basic psychological needs (competence, autonomy, and relationship with others) whose satisfaction increases intrinsic motivation and personal well-being (Huertas, 2012; Ames, 2013; Menéndez-Santurio and Fernández-Río, 2016). Autonomy refers to the willingness to experience the self as an agent, as the initiator of one’s own behaviors, being the origin of the perception or the source one’s own actions (Deci and Ryan, 2000). The relationship with others or *Relatedness*, refers to the desire to feel connected with others – loving and caring, and being loved and cared for (Deci and Ryan, 2000; Menéndez-Santurio and Fernández-Río, 2016). Competence is the need to feel effective when interacting with the environment or, as Deci and Ryan (2000) and Raimundi and Molina (2015) propose, the idea of experiencing opportunities by applying or expressing one’s own capacities. This theory links with the approach of Positive Action by young people whose maximum proponent was Hellison (1995, 2003, 2011).

Taking into consideration the approach of Positive Action Theory among young people (Seligman and Csikszentmihalyi, 2000; Lomas et al., 2016; Pawelski, 2016), various intervention programs have emerged over the last few decades which start by considering sports or physical activity as a way to achieve the objective of improving the personal and social development of teenagers thought to be ‘at risk’ (Escartí et al., 2010a,b). Although the aims and effectiveness of the programs have been varied, most of them have centered on increasing participants’ moral reasoning, attributions, self-concept, self-perception of efficiency, and the understanding of other people’s worlds. The Teaching Personal and Social Responsibility Model (Hellison, 2003) program is one of the most consistent. The model has been adjusted and applied in physical education classes in many countries (Hellison et al., 2000), including Spain (Escartí et al., 2013; Martinez et al., 2016).

Fishbein and Ajzen (1974, 1975, 1980) made an important contribution to the study of human behavior when they proposed the theory of reasoned action. Its importance is due to the complete research model that takes into account factors that are usually considered separately in other theories. Beliefs, for instance, are seen as behavioral aspects when they are specific for each subject or as regulations when they are considered relevant to groups and belonging. Other issues would be: attitudes are subjective standards, social norms or, in other words, objective dimensions of our normative beliefs, and intention toward the realization of a behavior. Alonso-Arroyo (2014) uses these factors to carry out research among second- and third-year students in secondary education (13–15 years). Similarly, Monsalvo (2012, 2013) ran a study with students during early years and elementary education, using methods that consider those four dimensions of action defined previously (beliefs, attitudes, social norms, intention) and applying them to the teaching units in the classroom in collaboration with the tutors. Results have led other authors (Carbonero et al., 2015) to analyze differences between academic performance and attitudes of personal and social responsibility within students of primary education.

In the recent years, new programs have appeared within the Spanish education system that attempt to work on the acquisition of habits of responsible education. Of note among them is the Botín Foundation’s project aimed at responsible education, specifically its Life and Values in Education program (LIVE Project; Argos et al., 2011; Melero and Palomera, 2011), and Prevent in Order to Live (POL) program. Both schemes are part of the line of work on responsible education that the Botín Foundation has applied among students in primary and secondary education.

A child’s style of moral behavior can be explained by taking their family background and home environment into consideration. The style of responsibility depends on the observation of others’ moral behavior, especially after observing the way their parents behave. Traditionally, family socialization has been considered an important factor of children’s psychosocial wellbeing and a basic theoretical construct in order to understand adjusted and adapted behaviors within society (Musitu and García, 2016). The evaluation of the process of socialization requires a theoretical perspective that conceptualizes how parents can influence their children. Some authors (Brody and Shaffer, 1982; Bandura, 2001; Diaz and Eisenberg, 2015) underline the role that parents play, as they act as a model of prosocial behavior and as models of moral restriction. In fact, studies have found evidence that those people who have observed prosocial models tend to be more prosocial than those who have never been exposed to them (López-Pérez et al., 2016). In addition, some studies have shown children whose parents have given them everything they ask for tend to yield more easily than children who are not exposed to this type of model (Brody and Shaffer, 1982). As a result it could be thought that the role model that a young person receives from their parents during childhood until preadolescence is fundamental and would orient a child’s moral behavior.

## Aims of this Study

The previous section introduced different programs that aim to work on attitudes of personal and social responsibility at school. Additionally, the role that family plays as a model for the acquisition of attitudes of this type has been discussed. Nevertheless, with the school setting in mind, there are other factors that can complete the process of acquiring personal and social responsibility, such as the importance of peers in the classroom. In this sense, responsibility explained through short stories can help emphasize the learning of moral judgment. Likewise, it is thought that tutorial action programs, coexistence plans, and schemes to promote reading might help to develop moral judgment among students. All of these share the idea of education in emotional judgment (Wray-Lake and Syvertsen, 2011) in order to strengthen character (Seligman and Petterson, 2004), which this research intends to study in the reading of short stories and folktales.

Specifically, this study aims to test the effectiveness of systematic training in social responsibility, involving different members of the educational community. In particular, it attempts to discover: (a) if the program produces significant improvements in social responsibility factors, as well as the reasoned action descriptors; (b) if, after applying the program, there are differences in the changes in social responsibility and reasoned action between males and females, (c) whether or not there is a relationship between factors of social responsibility or the descriptors of the reasoned action and parenting styles.

## Materials and Methods

### Participants

A total of 271 students (144 males and 127 females) in the first and second years of secondary education (12–14 years) from three different schools took part in the study. One school was randomly assigned as the experimental group and the other two as the control. Each class was taken to be a natural group, which meant that 132 students were assigned to the experimental group and 139 students belonged to the control group. All three schools are in urban areas and have intake from families from middle-range socioeconomic groups. The percentage of children coming from other countries was similar for all three schools (from 6 to 8%), mainly from Morocco, Rumania, and Ecuador. The demographic characteristics of all students were similar (see **Table 1**). Specifically, gender distribution was equally distributed (53% males in the experimental group and 52% in the control group). The students were grouped in the range of 12–14 years corresponding to the age of the academic level.

**Table 1:** Demographic characteristics of participants (*N* = 271).

	Experimental group^a^		Control group^b^	
Characteristic	*n*	%	*n*	%
Gender				
Male	71	54	73	52
Female	61	46	66	48
Age at time of survey (years)				
11	4	3	4	3
12	56	43	28	20
13	54	41	66	48
14	16	12	31	22
15	2	1	10	7
Number of brothers				
One	32	24	30	22
Two	77	59	77	55
Three	19	14	20	14
Four	4	3	7	5
Five or more	–	–	5	4
Family unit				
Father and mother	105	79	125	90
Mother	23	17	10	7
Others	2	2	3	2
Don’t know/don’t answer	2	2	1	1
Father’s highest education level completed				
Elementary school	36	27	32	23
Junior high school	35	27	52	37
High school	27	20	33	24
Bachelor’s degree or above	33	25	22	16
Don’t know	1	1	–	–
Mother’s highest education level completed				
Elementary school	42	32	17	12
Junior high school	39	29	51	37
High school	17	13	31	22
Bachelor’s degree or above	34	26	40	29

*^a^*n* = 132; ^b^*n* = 139.*

The study was conducted in accordance with the 1964 Helsinki declaration and its later amendments or comparable ethical standards, with the approval of the management boards of the schools. Ethical approval was not required for this study in accordance with the national and institutional requirements. Participation in the study was voluntary. Written informed consent was obtained from the parents/legal guardians of all participants.

### Measures

#### Assessment Scale of Social Responsibility Attitudes in Secondary Education (Otero, 2015)

This scale measures several aspects of social responsibility of students in compulsory secondary education. A five-option Likert scale was used, which ranged from 1 (*not/never*) to 5 (*yes/always*). The survey had a total of 35 items grouped into ten factors that account for 55% of total variance, and the reliability indices in the study were 0.53 < α < 0.79. These factors are: (a) Respectful with the Context (five items, α = 0.54), (b) Friendly and Willing to Help (four items, α = 0.69), (c) Self-discipline and Perseverance (seven items, α = 0.70), (d) Acceptance of Errors (two items, α = 0.79), (e) Well-mannered, Friendly and Tidy (three items, α = 0.55), (f) Commitment (four items, α = 0.64), (g) Obedience (two items, α = 0.78), (h) Consistent, Prudent, and Self-controlled (four items, α = 0.53), (i) Parents as Model of Socially Responsible Behavior (two items, α = 0.72), and (j) Parents as Model of Perseverance (two items, α = 0.72).

Bearing in mind the criteria established by the theory of reasoned action and the contributions from Otero (2015), this scale also measures six descriptors of reasoned action while working with the program: (a) Beliefs (three items, α = 0.65), (b) Attitudes (six items, α = 0.72), (c) Standards (nine items, α = 0.78), (d) Intentions (seven items, α = 0.78), (e) Habits (four items, α = 0.61), and (f) Models (seven items, α = 0.67).

#### Parent–Adolescent Communication Scale (PACS, Barnes and Olson, 1982; Translated by Musitu et al., 2001)

The questionnaire has two scales aimed at teenagers and measures communication between children and mother, and children and father. Each scale has 20 items in a Likert-type format with five values: from 1 (*never*) to 5 (*always*). In the Spanish version, the scale shows a structure of three factors for mothers and fathers separately: (a) Open Communication (11 items, α = 0.87). (b) Offensive Communication (4 items, α = 0.76), and (c) Avoidant Communication (5 items, α = 0.75).

### Procedure

In order to carry out the research inside a school, it was mandatory to obtain permission from the school and the educational authorities, as well as the informed consent from all the families. The study was conducted over a full 2-year period. The educational material was prepared during the first year: the team selected all the stories and created the reading plan and the working plan to develop beliefs, attitudes, norms, intentions, habits, and models. During the second year, the responsibility attitudes questionnaire and family communication questionnaire were applied, and individualized student monitoring was designed, with mentoring sessions in order to train students and their families. Finally, the application of the questionnaires and the evaluation of the interviews that tutors conducted with their students took place at the beginning and at the end of the academic year. The educational program follows the methodology proposed in the theory of reasoned action (Fishbein and Ajzen, 1975), and Otero’s (2015) proposal was added to it, including habits and models to work with the students (see **Table 2**).

**Table 2:** Characteristics and structure of the program.

Objective	Recipients	Procedure
Beliefs	Students	• Educational program: Let’s think: emotional judgment and good decisions
Standards
Social responsibility
Standards	Students	• Self-evaluation about your responsibility social applied in mentoring individualized
Attitudes	Students	Mediator-lector student participates in:
Intentions		• Leading sessions with the didactic units
		• Supervises school magazine
		• ‘The used book market’
		• Blogs
Models	Faculty	Training courses:
Habits
		• The student as mediator-lector and its role for the integration of the *Plan de Fomento de la Lectura* (PFL, *in English, Reading Promotion Plan*) and Plan de Convivencia (*in English, Coexistence Plan*)
		• Intercultural stories for the teaching human rights
	Family	• School for parents: understanding of the value of the responsibility
		• Reading workshop
		• Participation in the blogs

In order to work with *beliefs*, a teaching program called “Playing to think: emotional judgment and good decisions” was developed (See Appendix, **Table A1**). It consists of 14 teaching units, each focussing on one story. The 14 stories were selected from a total of 71 taken from the traditional literature bearing in mind the students’ age range. The selection criteria were: (a) teachers’ preferences, (b) stories that work on several factors of social responsibility, (c) well-known authors from universal or children’s literature or traditional tales, and (d) stories known and adapted by students’ families in workshops for parents held at the school. Each story begins with a series of features that explain or define descriptors of responsibility that the program intends to work on. Secondly, there was a statement of the objectives of those elements that involve not only reasoned action (such as beliefs, rules, and attitudes) but also habits and models (for instance, emotional issues such as identification with the characters leading to the projection of the elements as those involved in intentions). After that, several activities were presented. These activities comprised a series of questions seeking reader identification with the main character in the story (describing). Furthermore, the plot and all the conflicts that appear in the story were turned into a moral laboratory (narrating). This was an opportunity for the children to test their decisions, and learn to take responsibility about their possible consequences (projecting). All teaching units ended with self-assessment: the student’s self-reflection and self-recognition in story, enhancing the narrative reflection that is suggested in the introduction section. All questions refer to a student’s prosocial, emotional, and cognitive aspects. This program includes a guide for the teacher who applies it.

Personal interviews were used by the tutors to work with the students on the norms. Tutors were suitably trained to work on the program. Orientation sessions prepared them to develop, implement, and monitor the program. An observation questionnaire was developed for each student to fill out while s/he was individually tutored and it was followed up over the whole course by the tutor with the approval of the parents.

In order to work with attitudes and intentions, the figure of the mediator–instructor student was created. These students worked on collaboration in implementing the program, especially when designing and applying activities related to social responsibility and those based on the theme of the stories that students were working on. The main objective was to identify whether or not students choose the beliefs of the people closest to them when there is a conflict between their behavioral beliefs, their convictions, the stories they work with and the beliefs assigned to those significant others (parents, teachers), as stated by Fishbein and Ajzen’s (1975) model.

The only way to avoid this dissonance was through individualized tutoring, as mentioned above, but also through the peer group. In this case the role of the mediator–instructor was highly relevant, due to their role as a guide and as a leader to follow up the teaching unit activities about the stories. The mediator–instructor student was trained to acquire communicational skills such as dialog and peaceful resolution of conflicts. Therefore, the role of the mediator–instructor student was closely related to the students mediating coexistence. The coexistence plan included a group of training activities for the mediator students that could also be used with their mediator–instructor peers.

Tutors and parents were trained to work with the models and habits. Tutors were trained at meetings with the School counselor and parents attended sessions of the ‘*parents’ school*’ held at the experimental group’s center to work on the main issue of the value of responsibility.

### Data Analysis

To assess the effectiveness of the program, we calculated the differences between the scores from the pretest and the posttest measures in the control and the experimental group. For paired comparisons, the *t-*test for two independent groups was used, including Cohen’s *d* effect size (Cohen, 1988), considering: *d* = 0.20 small, *d* = 0.50 medium, and *d* = 0.80 large effect size. For this purpose, we used the statistical package IBM SPSS Statistics, version 23 (2015). All statistical analyses used showed a 95% confidence level. The Pearson correlation coefficient was used in order to measure strength of linear correlation between the variables of parental styles and the statements issued by the students about the benefits that they believe the program has as result.

## Results

### Effects of Applying the Educational Program

Taking into consideration the factors of social responsibility, scores in all variables are higher in the experimental group than in the control group (see **Table 3**), an independent-samples *t*-test indicated scores in self-discipline were significantly higher for the experimental group (*M* = –0.06, *SD* = 2.90) than for the control group (*M* = –2.29, *SD* = 6.83), *t*(269) = 3.46, *p* = 0.001, with a size of low effect *d* = 0.42. It has been observed that scores from the experimental group are kept at the initial levels, while the scores from the control group decrease considerably. A similar effect has been observed with the Commitment factor, with higher scores for the experimental group (*M* = 0.17, *SD* = 2.28) than the control group (*M* = -0.66, *SD* = 4.04), *t*(269) = 2.07, *p* = 0.039, also with a low effect size, *d* = 0.25.

**Table 3:** Differences the pretest to the posttest measures when evaluating social responsibility and descriptors of the action reasoned between the experimental group and the control group.

	Experimental group^a^		Control group^b^				Cohen’s
Measure	*M*	*SD*	*M*	*SD*	*t*(269)	*p*	*d*
Respect	0.03	2.64	-0.53	4.04	1.36	0.176	0.16
Friendly	0.04	1.43	0.03	3.60	0.27	0.978	0.03
Self-discipline	-0.06	2.90	-2.29	6.83	3.46	0.001	0.42
Acceptance of errors	0.25	1.55	-0.16	2.50	1.62	0.106	0.20
Well-mannered	-0.21	1.76	-0.53	2.44	1.23	0.221	0.15
Commitment	0.17	2.28	-0.66	4.04	2.07	0.039	0.25
Obedience	0.34	2.23	0.17	2.91	0.54	0.591	0.06
Self-control	0.33	2.24	-0.16	3.45	1.38	0.170	0.17
Family: model of social responsibility	0.27	2.44	0.05	3.04	0.66	0.510	0.08
Family as a model of perseverance	-0.36	2.52	0.01	3.19	-1.07	0.285	-0.13
Beliefs	-0.25	1.99	-0.45	3.12	0.64	0.523	0.08
Attitudes	-0.04	3.08	-1.27	4.86	2.46	0.014	0.30
Standards	0.65	3.09	-0.21	7.04	1.29	0.199	0.16
Intentions	-0.13	2.95	-1.89	5.58	3.23	0.001	0.39
Habits	0.31	2.44	-0.38	4.61	1.52	0.129	0.18
Models	-0.11	4.12	0.23	4.83	-0.64	0.523	-0.08

*^a^*n* = 132; ^b^*n* = 139.*

Additionally, the gains in the descriptors of reasoned action are higher in the experimental group when compared with the control group. An independent-samples *t*-test indicated that scores in attitudes were significantly higher for the experimental group (*M* = –0.04, *SD* = 3.08) than for the control group (*M* = –1.27, *SD* = 4.86), *t*(269) = 2.46, *p* = 0.014, with a low effect size, *d* = 0.30. A similar effect has been identified with the intentions factor, with higher scores for the experimental group (*M* = –0.13, *SD* = 2.95) when compared to the control group (*M* = –1.89, *SD* = 5.58), *t*(269) = 3.23, *p* = 0.001, also with a low effect size, *d* = 0.39. Regarding the other variables, there is no other significant difference.

It is interesting to discover whether or not there are differences between genders in the level of social responsibility after being involved in the educational program (see **Table 4**). Such differences exist in the factor of Well-mannered, Friendly and Tidy, with higher scores for males (*M* = 0.09, *SD* = 1.54) in comparison with females (*M* = –0.54, *SD* = 1.96), *t*(130) = –2.02, *p* = 0.045, with a low effect size, *d* = –0.35. There is no difference between males and females within the descriptors of reasoned action.

**Table 4:** Differences in the pretest to the posttest measures when evaluating social responsibility and descriptors of the action reasoned among male and female within the experimental group.

	Male^a^		Female^b^				Cohen’s
Measure	*M*	*SD*	*M*	*SD*	*t*(130)	*p*	*d*
Respect	0.07	2.55	-0.01	2.78	0.18	0.855	0.03
Friendly	-0.08	1.36	0.19	1.51	-1.13	0.261	-0.20
Self-discipline	-0.87	2.70	-0.04	3.16	-0.09	0.921	-0.01
Acceptance of errors	0.11	1.25	0.42	1.84	-1.15	0.253	-0.20
Well-mannered	0.08	1.54	-0.54	1.96	-2.02	0.045	-0.35
Commitment	0.21	2.25	0.12	2.34	0.22	0.823	0.04
Obedience	0.66	2.07	-0.03	2.37	1.79	0.076	0.31
Self-control	0.33	2.48	0.33	1.95	-0.10	0.992	-0.02
Family: model of social responsibility	0.12	2.54	0.44	2.33	-0.76	0.449	-0.13
Family: model of perseverance	-1.05	3.26	-0.86	3.59	-0.32	0.747	-0.06
Beliefs	-0.33	2.01	-0.15	1.99	-0.50	0.616	-0.09
Attitudes	0.35	2.69	-0.51	3.44	1.61	0.109	0.28
Standards	0.90	3.06	0.34	3.13	1.05	0.297	0.18
Intentions	-0.19	2.17	-0.04	3.68	-0.29	0.772	-0.05
Habits	0.25	2.35	0.37	2.56	-0.27	0.790	-0.05
Models	-0.28	3.94	0.08	4.33	-0.51	0.911	-0.09

*^a^*n* = 71; ^b^*n* = 61.*

### Relationship with Parenting Styles

It has been observed that an open parental style is more positively correlated with variables of personal and social responsibility (see **Table 5**). It should be pointed out that there is high and positive correlation between respect for a mother’s open parental style, *r*(130) = 0.42, *p* < 0.001, in comparison with a father’s style, *r*(130) = 0.40, *p* < 0.001, self-discipline and a father’s open style, *r*(130) = 0.40, *p* < 0.001, and commitment to a father’s open style, *r*(130) = 0.40, *p* < 0.001. On the other hand, although to a lesser extent, respect also positively correlates with an offensive parental style, both for the mother, *r*(130) = 0.30, *p* < 0.01, and for the father, *r*(130) = 0.21, *p* < 0.05. Finally, it should be noted that there is a positive correlation between being friendly and an avoidant maternal style, *r*(130) = 0.19, *p* < 0.05.

**Table 5:** Significant correlation factors of social responsibility, descriptors of the action reasoned and parental styles.

Measure	Mother open	Mother offensive	Mother avoidant	Father open	Father offensive	Father avoidant
Respect	0.42***	0.30**	-0.09	0.40***	0.21*	-0.07
Friendly	0.10	-0.06	0.19*	0.08	0.01	0.12
Self-discipline	0.31***	0.23*	0.03	0.40***	-0.17	0.05
Acceptance of errors	0.34***	–	0.11	0.33**	-0.09	0.14
Well-mannered	0.10	0.06	0.14	0.12	0.09	0.10
Commitment	0.23*	-0.17	0.09	0.40***	-0.10	0.09
Obedience	0.09	-0.12	0.08	0.29***	0.02	0.07
Self-control	0.22*	-0.10	0.18	0.21*	-0.08	0.09
Family: model of social responsibility	0.02	0.05	0.06	0.16	0.13	-0.02
Family: model of perseverance	0.17	-0.05	-0.06	0.14	0.03	-0.02
Beliefs	0.15	-0.13	0.14	0.12	-0.09	0.09
Attitudes	0.22*	-0.07	0.11	0.23*	-0.03	0.07
Standards	0.25**	0.21*	0.03	0.37***	-0.05	0.02
Intentions	0.33***	-0.13	0.21*	0.35***	-0.02	0.24*
Habits	0.37***	0.25**	-0.09	0.37***	0.19*	-0.09
Models	0.22*	-0.11	0.00	0.34***	-0.01	-0.02

*^∗^*p* < 0.05, ^∗∗^*p* < 0.01, ^∗∗∗^*p* < 0.001.*

As with the factors of social responsibility, the open parental style positively correlates with the descriptors of reasoned action, except with the factor of beliefs. Worth noting is the high and positive correlation between a father’s open style and standards, *r*(130) = 0.37, *p* < 0.001, and a mother’s open style and intentions, *r*(130) = 0.33, *p* < 0.001, a father’s open style and intentions, *r*(130) = 0.35, *p* < 0.001, a mother’s open style and habits, *r*(130) = 0.37, *p* < 0.001, and a father’s open style and habits, *r*(130) = 0.37, *p* < 0.001. With less intensity, however, there is a positive correlation between a mother’s offensive style and rules, *r*(130) = 0.21, *p* < 0.05. On the other hand, a relationship seems to exist between the offensive parental style and habits, since a positive correlation between an offensive mother’s style and habits, *r*(130) = 0.25, *p* < 0.01, has been identified, as well as a relationship between an offensive father’s style and habits, *r*(130) = 0.19, *p* < 0.05. A similar effect appears between avoidant parental style and intentions. An avoidant mother’s style positively correlates with intentions, *r*(130) = 0.21, *p* < 0.05, and an avoidant father’s style also positively correlates with intentions, *r*(130) = 0.24, *p* < 0.05.

### Assessment of the Program

Students positively assessed the intervention program. Overall, their opinions are that the program: (a) has improved their social responsibility, (b) they like those peers who are more polite, (c) teachers appreciate that their own behavior is friendlier, (d) participants feel they will be more steadfast and committed when behaving and doing their tasks, (e) they feel that they pay more attention and are more engaged, (f) they believe their parents have seen them improving in coexistence, (g) they believe that they have had an overall improvement in all aspects of their lives, (h) they dislike it when teachers argue, and (i) they have improved in order and organization, which is the most valued claim among the students from the experimental group. The majority of these statements positively correlated with aspects that have been trained in the program (see **Table 6**). Intentions correlated with most of the above statements. On the other hand, it should be highlighted that there is a high correlation between perseverance and intentions, *r*(130) = 0.45, *p* < 0.001, perseverance and models, *r*(130) = 0.39, *p* < 0.001, and being more attentive and committed with habits, *r*(130) = 0.38, *p* < 0.001.

**Table 6:** Significant correlations of students’ claims about the benefits of the program and the descriptors of the action reasoned.

Measure	Beliefs	Attitudes	Standards	Intentions	Habits	Models
Improving responsibility	0.06	0.09	0.06	0.21*	0.34**	0.29**
Preference for kind classmates	0.17	0.22*	0.21*	0.36***	0.16	0.06
Teachers who appreciate to be friendly	0.08	0.22*	0.27**	0.35***	0.21*	0.27**
You will be more persistent	0.23*	0.30**	0.16	0.45***	0.31**	0.39***
More attentive and committed	0.03	0.21*	0.08	0.26**	0.38***	0.05
Your father have seen you improving in coexistence	0.13	0.17	0.09	0.22*	0.23*	0.25**
Your mother have seen you improving in coexistence	0.13	0.20*	0.13	0.38***	0.29**	0.22*
You think that you have generally improved	0.09	0.05	0.08	0.16	0.14	0.20*
You hate teachers who argue	0.19*	0.16	0.01	0.13	0.05	0.00
You have improved in organization and order	0.29**	0.32**	0.17	0.28**	0.27**	0.22*

*^∗^*p* < 0.05, ^∗∗^*p* < 0.01, ^∗∗∗^*p* < 0.001.*

## Discussion

This research studied the effectiveness of a program for improving attitudes of personal and social responsibility in the first and second year of secondary education, using individualized sessions and taking into account gender, parental styles, and personal relationships. Students who have followed the program show higher scores in most of the analyzed variables when compared to those that have not been involved. It has also been observed that in two of the reasoned action descriptors they scored significantly higher. Additionally, participants obtained significant scores in those factors of social responsibility related with commitment, self-discipline, and perseverance.

Along similar lines, Deci and Ryan (2000) argue that at this age intrinsic motivation is still in the acquisition process. As a result, teenagers continue depending on their models, especially regarding habits and intentions. However, these intentions are the result of acceptance of the standards of their peers and not their attitudes and acceptance of standards internalized by them. In fact, obedience toward their parents is shown in order to avoid parental dissatisfaction. It can be concluded that during the ages studied (12–14 years old) students are still in the process of acquiring autonomy, meaning that in this period students are still understanding that their responsibility is based on obedience and reliability with their figures of attachment, and not on criteria of autonomy and/or self-efficacy.

Several studies have obtained similar results and conclusions, such as research conducted by Maliki et al. (2010), Wray-Lake and Syvertsen (2011), and Salusky et al. (2014) who interviewed teenagers regarding their responsibility criteria. The majority of participants said that they meet the standards because they consider it necessary for coexistence. However, other participants pointed out that they do so by obligation and to avoid punishment. Considering these ideas, the definition of the concept of responsibility for these teenagers seems to be associated with compliance. In a similar vein, Alonso-Arroyo’s (2014) study with 14- to 16-year-old students aimed to rate the effectiveness of a volunteering program and concludes that there is no improvement in participants’ altruistic attitudes.

In this regard, the anthropological studies conducted by Ochs and Izquierdo (2009) and Ochs (2011) find that children’s participation in domestic activities not only provides practical skills, but also promotes moral responsibility, thus creating social awareness, responsiveness to the needs of others and self-sufficiency. In terms of moral development, the cultures observed by these researchers had already acquired personal and social responsibility at the age of 12 years. Such moral awareness creates in children a sense of belonging to the group in which they are involved, which in turn was expressed in a shared identity. Participation in domestic activities allows their relationship and identification with the family and the community (Paradise and De Haan, 2009) to be strengthened. Child participation in these activities was associated with their general well-being and a sense of identity within their social group, which helps to understand the positive opinions about work observed among the children taking part in the research (Ames, 2013).

It might be that the problem facing our society is linked to our culture, in which children increasingly delay the acquisition of attitudes of responsibility related with autonomy and social solidarity. This might be the result of a sense of overprotection and avoiding or not giving them domestic responsibilities as individuals. Awareness of the idea of the needs of others (Peters, 2015; Caba-Collado et al., 2016; Menéndez-Santurio and Fernández-Río, 2016) and involvement in meeting them creates awareness of belonging and therefore moral awareness, which according to recent evolutionary studies seems to be changing (Galo, 2016; Medina-Vicent, 2016). In this regard, the program presented here seems to promote, through fiction, identification with the needs of others and thus promote moral responsibility and social consciousness. In addition, training in decision-making and in the projection of consequences generates self-motivation, confidence in one’s own capacity of response to other people’s needs and the independence of our moral judgments. One of the limitations of the present research lies in transferring the skills to real life and for specific tasks.

Taking the gender variable into consideration, higher scores have been identified among males after completing the program. However, these significant statistical differences are observed only in the social responsibility factor when related with being well-mannered, friendly and tidy. The results of the LIVE Project (Argos et al., 2011; Melero and Palomera, 2011) show greater efficiency within males, especially concerning the emotional aspects and assertiveness. Nevertheless, other research highlights higher scores among females in autonomy and responsibility when compared to males (Martinek et al., 2006; Monsalvo, 2012; Alonso-Arroyo, 2014).

In terms of communicative styles, the results show that an open parental style, both from the mother and the father, positively correlates with improvements in personal and social responsibility within students in the experimental group. Their moral autonomy and prosocial behaviors are clearly improved according to the judgments issued by the students; this is in line with results from Diaz and Eisenberg (2015) and Musitu and García (2016) when studying the acquisition of this process. Furthermore, these variables validate the model and habits variable, introduced as a contribution from this study in the reasoned action methodology (Otero, 2015). However, there is also a relationship that should not be forgotten: the link between respect and offensive parental style, possibly due to a low degree of personal autonomy, which is still in its development phase.

## Conclusion, Limitations, and Future Directions

Considering the results, it can be concluded that effort focussed on improving personal and social responsibility, in the individualized action of the tutor, has been beneficial for students in the first two courses of secondary education. Students suggest that they have improved significantly in responsibility skills due to the program model they have been involved in. The most significant results are identified among the variables of self-discipline, commitment, attitudes, intentions, and are closely related to an open parental style.

This transversal study focuses on a specific evolutionary period, which on one hand represents a limitation, but on the other helps to make the results more understandable. If students belonged to higher courses, results might be different, especially regarding obedience and respect given their higher degree of autonomy, which would reduce them being associated to the familiar environment, and/or to greater self-reliance. Consequently, it would be convenient to study another age range to complement the one studied here, and carry out longitudinal studies that allow the evolution of social and personal responsibility to be observed.

Additionally, the action of the tutors has been effective according to students’ self-reports. The instruction process should be applied repeatedly, aiming to improve the model and the habits. In subsequent research it would be interesting to collect reports from tutors about how students improve in terms of responsibility when applying the program. These observed-reports would complement the results of the students’ self-reports. Similarly it would be suitable to study in depth the influence of gender as a factor of personal and social responsibility and the reasoned action descriptors. It is understood that variables of socio-emotional development might condition its acquisition, but no study investigates this aspect. Consequently, there is a need for more studies that investigate emotions, with the emotional trials. In this regard, new measurements that study the relations between self-assessment and teaching units, as well as other kinds of reports should be used. It is also necessary to take a more detailed look at the model and how social responsibility is measured so that the questionnaire includes more reliable indices. In addition, systematic coaching and ongoing monitoring by tutors at this level is also a pending task. The task of the teacher or tutor has been especially well valued by students. Teachers should improve the orientations toward better emotional judgment among students, and, additionally, they should offer feedback regarding their students and provide them with proposals in order to solve any difficulties that might arise. All this would mean intensive training for tutors, which should be integrated within the schedule for organizing and designing the school curriculum. Similarly, it should be pointed out that the training should not only be for the staff in charge of implementing the program, but for the entire educational community and their families.

## Author Contributions

All authors made substantial contribution to the theoretical framework, design, data collection or interpretation of this study. All authors approved the final manuscript as submitted.

## Conflict of Interest Statement

The authors declare that the research was conducted in the absence of any commercial or financial relationships that could be construed as a potential conflict of interest.

## Appendix

**TABLE A1:** Structure, traits of personality and social responsibility factors worked with the training program: Let’s play to think: the emotional judgment and good decisions.

Unit	Traits of responsibility	Factors
I am Sam	(1) Expectative from parents and children, (2) parents as model of social responsibility, (3) parent models of perseverance and self-discipline.	F9 and F10
The day of the responsibility	(1) Respect toward nature and classroom materials., (2) respect at home.	F1
The box of chocolates	(1) Empathy and “listening,” (2) Fellowship, (3) get along with adults, (4) be collaborative, (5) capacity for coexistence.	F2
The disobedient Prince	(1) Homework done, clean notebook, complete, etc. (2) attention to the teacher, punctuality, (3) participation in housework.	F3 and F7
The village of unclean people	(1) Civility and personal hygiene, (2) Organization and order.	F5
The child and the bomb	(1) Self-attribution od errors and acceptation of its consequences with self-criticism, (2) consistent, prudent and self-control.	F4 and F8
A girl very superior	(1) Honesty, (2) assist and collaborate with coworkers, (3) generosity, against selfishness.	F6
A very busy parents	(1) A model of social responsibility and perseverance is a model of parents dedicated to their children.	F9 and F10
Household tasks	(1) Respect in the family context, (2) order in the family context.	F1
The last leaf	(1) Well-mannered, (2) be friendly and be ready to the support and to collaborate.	F2
The messed fairy	(1) Friendliness and well-mannered, (2) order the tasks and homework; (3) collaboration in the household tasks.	F5
The Gnome	(1) Being self-disciplined and constant worker, (2) bring the made activities, clean notebook, full, etc., (3) record in the Studio, (4) obedience; (5) comply with what it promises.	F3 and F7
The king who makes deserts	(1) The importance of controlling own emotions, (2) the importance of accepting errors, (3) justice as a commitment to each other.	F4 and F6
Lost in the forest	(1) Self-evaluation and recognition of errors to amend them, (2) learn to control temper tantrums and mood, (3) value patience as a way to achieve our goals and enhance friendship.	F8

*F1 = Respectful with the Context; F2 = Friendly and Willing to Help; F3 = Self-discipline and Perseverance; F4 = Acceptance of Errors; F5 = Well-mannered, Friendly and Tidy; F6 = Commitment; F7 = Obedience; F8 = Consistent, Prudent and Self-controlled; F9 = Parents as Model of Social Responsibility Behavior; F10 = Parents as Model of Perseverance.*
